# A Rare Case of Neurosyphilis with Calvaria Osteitis Presenting in Pregnancy

**DOI:** 10.1155/2023/8856775

**Published:** 2023-12-19

**Authors:** Gisella M. Newbery, Christine E. Henricks, Julie A. Vircks, Andreina Colina, David C. Mundy

**Affiliations:** ^1^University of Missouri-Kansas City School of Medicine, 2411 Holmes Street, Kansas City, MO 64108, USA; ^2^University Health, 2301 Holmes Street, Kansas City, Missouri 64108, USA

## Abstract

**Background:**

The incidence of syphilis throughout the world is increasing. Rates in pregnancy are similarly rising, presenting risks of an untreated syphilis infection that can be detrimental to the mother and fetus. Although routine screening for syphilis infections is recommended at the initial prenatal visit, there is a lack of universal agreement on rescreening pregnant people and approximately 50% of syphilis cases are asymptomatic in the general population. Furthermore, some symptoms of syphilis can overlap with nonspecific pregnancy-related symptoms. Meanwhile, Treponema pallidum can spread to various maternal and fetoplacental tissues quickly after infection and occur at any stage of syphilis.

**Case:**

A 26-year-old gravida 5 para 2 presented with a new onset headache and visual and auditory changes at 23 weeks of gestation. A computerized tomography scan revealed numerous ill-defined lytic lesions throughout the calvarium, suspicious for syphilitic osteitis. She tested positive for syphilis antibodies with a rapid plasma reagin (RPR) titer of 1 : 32. Cerebrospinal fluid evaluation from a lumbar puncture resulted in reactive fluorescent treponemal antibody (FTA) testing. She was diagnosed with secondary syphilis with osteitis and neuro and otic components. She completed 14 days of intravenous aqueous crystalline penicillin G with additional benzathine penicillin G 2.4 million units intramuscular weekly for two weeks. There was no evidence of congenital syphilis on neonatal examination.

**Conclusion:**

Syphilitic osteitis and neuro, otic, or ocular syphilis infections occur rarely in the nonpregnant population, and therefore, little data in pregnancy is available to inform outcomes in these specific disease states. It is of paramount importance to complete appropriate syphilis screening, recognize symptoms, and consider utilizing rescreen protocols to ensure prompt infection identification and treatment. For neuro, otic, and ocular syphilis, aqueous crystalline penicillin G (as opposed to benzathine penicillin G) is required to achieve treponemicidal concentrations in those physiologic compartments. There is no agreement as to the appropriate treatment regimen for the rare finding of syphilitic osteitis.

## 1. Introduction

Syphilis infections worldwide continue to rise at an alarming rate. According to the World Health Organization, approximately one million pregnant people are infected with syphilis annually [1–3]. Treponemal infections in pregnancy present a dual threat: short- and long-term maternal sequelae of an untreated infection and vertical transmission to the fetus resulting in congenital syphilis [1, 2, 4]. Congenital syphilis occurred in 661,000 births in 2016 globally and represents a significant cause of stillbirth, neonatal deaths, and preterm or low birth weight infants worldwide [5]. Pregnancy represents a unique intersection of increased healthcare access for many, including routine infectious disease testing. Due to the high-risk nature of syphilis infections with an effective treatment available, early screening for syphilis is recommended in pregnancy. Repeat testing in high-risk populations or in high prevalence areas is recommended later in gestation (28 weeks of gestation and delivery), but late gestation testing is not universally mandated [4, 6, 7].

Approximately fifty percent of all syphilitic infections are asymptomatic, which is problematic for the identification of an early infection that could occur in pregnancy after initial testing is completed. During this time, Treponema pallidum can disseminate within days of initial infection. The bacterium can infect most human tissue, including the fetoplacental unit and the maternal central nervous system [8, 9]. In those who do experience a symptomatic syphilis infection, the clinical symptomatology can be vague. Known as the “great imitator,” syphilis infections can cause a vast array of symptoms including headache, malaise, appetite changes, and gastrointestinal symptoms including nausea. These symptoms may overlap with nonspecific symptoms frequently seen in pregnancy [10].

We present a case of a persistent headache in the second trimester of pregnancy resulting from a syphilis infection that developed in the pregnancy interval, with the very rare finding of maternal calvarial lytic lesions on computerized tomography (CT) in conjunction with neuro and otosyphilis.

## 2. Case Presentation

The 26-year-old gravida 5 para 2 initiated prenatal care at 7-week gestational age (GA). Her history was pertinent for previous polysubstance use (methamphetamines, cannabis), but negative for infectious disease, including a nonreactive syphilis antibody.

**Figure 1:**
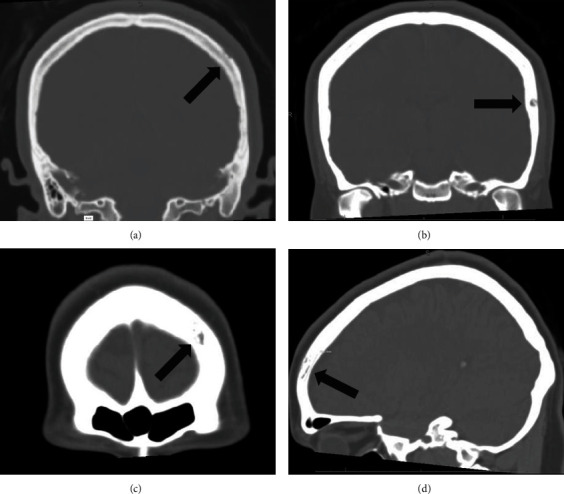
Multiple ill-defined lytic/permeative lesions (arrows) throughout the calvarium, several of which involve the outer table of the calvarium (a–d). The largest ill-defined lytic osseous lesion measured approximately 2 cm, with slight depression of the outer table and underlying soft tissue compartment (d).

At 23-week GA, the patient reported a one-month history of a headache, which the patient initially ascribed to a very minor incident of hitting her head. She also described a constant auricular aura consisting of muffled noises in addition to blurry vision. A head CT was ordered which revealed numerous ill-defined lytic lesions throughout the calvarium (Figure 1). Lab screening demonstrated an RPR with a 1 : 32 titer. Routine HIV screening was also performed at this time, which was negative.

Given the constellation of symptoms in conjunction with lytic calvarial lesions on CT and positive syphilis antibody testing, the patient was admitted for further evaluation. Infectious disease specialist colleagues were consulted, and they arrived at a diagnosis of secondary syphilis. The consultants recommended treatment with intravenous (IV) aqueous crystalline penicillin (PCN) G 4 million units (MU) every 4 hours, as opposed to the intramuscular PCN treatment utilized for secondary syphilis alone, due to additional findings concerning for neuro and otosyphilis with calvarial osteitis. Neurology and ophthalmology were also consulted, and their exams were notable for sensorineural hearing loss in the right ear and no syphilitic ocular findings, respectively. A detailed fetal ultrasound was performed revealing no gross fetal abnormalities and an appropriately grown fetus for gestational age.

Further investigation with a fluoroscopically guided lumbar puncture (LP) was performed due to the patient's headache and calvarial lesions. The cerebrospinal fluid (CSF) was Gram stain negative with additional bacterial and viral cultures also negative. The CSF BioFire (bioMérieux, Salt Lake City, Utah), which provides a rapid preliminary result, was negative, and the CSF treponemal antibody and RPR were sent for evaluation to an outside laboratory. Infectious disease consultants recommended the continuation of IV aqueous PCN G until the final CSF results were received.

The final CSF evaluation result returned several days later with a nonreactive venereal disease research laboratory (VDRL) and a reactive FTA, the latter diagnostic of neurosyphilis. Infectious disease consultants recommended a minimum of 14 days of IV aqueous crystalline (PCN G at 4 million units every four hours) per Centers for Disease Control and Prevention guidelines [11] with additional benzathine PCN G 2.4 MU intramuscular (IM) weekly for two weeks. The patient was discharged after the aqueous crystalline IV PCN G was completed and presented to the outpatient clinic for the remaining IM doses.

The patient was readmitted for delivery at 37-week GA due to a nonreassuring nonstress test. The repeat maternal RPR titer had decreased to 1 : 8. She delivered a male infant via cesarean delivery with APGAR scores of 8 and 9 at 1 and 5 minutes, respectively, weighing 3,325 grams. There was no evidence of congenital syphilis on the newborn physical exam. A neonatal RPR was obtained at birth followed by the administration of 1 dose of 165,000 units IM PCN G. Neonatology intensive care unit observation was planned if the infant's titer was more than fourfold greater than the recent maternal titer of 1 : 8 [12]. The infant's RPR titer was 1 : 4, indicating adequate maternal treatment.

Regarding follow-up, the maternal RPR titer will be repeated at 6 months from the time the initial titer was drawn, with an expected 4-fold decline indicating adequate treatment [12]. A head MRI demonstrated resolution of the calvarial lytic lesions 4 months after the initial diagnosis. Beyond routine pediatric follow-up, the infant follow-up was planned at 2-3 months with a repeat RPR titer and thorough examination, the results of which are unavailable.

## 3. Discussion

Headaches in pregnancy present a unique diagnostic challenge fraught with cautiousness regarding imaging, pharmacotherapy, and diagnostic procedures due to the pregnancy state. Although most headaches in pregnancy are benign, infectious etiologies can be a rare source of a persistent headache but may be a consideration given the rising incidence of syphilis [13, 14]. This is the first known case report of lytic lesions of the maternal calvarium due to a syphilis infection, with accompanying neuro and otosyphilis, that developed during the pregnancy.

Calvaria involvement in syphilis rarely occurs in the nonpregnant population and is reported in less than 1% of infections. Osteitis is typically most likely to occur in tertiary or congenital syphilis. In a systematic review published in 2014, the authors only found 36 cases of syphilitic osteitis over the last 50 years specifically in secondary syphilis, with 57% of those affecting only the skull. Seventy-three percent of syphilitic bone lesions, however, were multifocal (long bones, spine, rib, clavicle, and sternum) and noted to be osteolytic in 51% of cases [15]. Notably, the median RPR with syphilitic osteitis was 1 : 64 in this study sample. All patients who received treatment had resolution of their bone pain with antibiotic treatment, and most had resolution of the lesions after several months; however, the optimal treatment duration or type of penicillin has not been ascertained [15]. The differential diagnosis for multiple lucent calvarial lesions remains broad, however, and can include lytic metastases, multiple myeloma, leukemia, sequela of osseous infection, neurosarcoid, or the Langerhans cell histiocytosis. Follow-up imaging should be obtained for the resolution of these lytic lesions, and the lack of resolution should prompt further imaging and a biopsy.

Similarly, neurosyphilis is also considered a rare finding in the general population occurring in 1.8% of all syphilis infections but is not well described specifically in pregnancy [16]. It is important to recognize that neurologic involvement can occur during any stage of syphilis and can be either asymptomatic or symptomatic [8, 17]. It is necessary for all patients, including those who are pregnant, who have neurologic signs or symptoms with a positive serum syphilis test to undergo an LP for CSF evaluation to determine the appropriate treatment plan [18]. Additionally, otic and ocular syphilis are distinct entities from neurosyphilis, can occur during any stage of syphilis, and may occur in concert with neurosyphilis [11].

Neuro, otic, and ocular syphilis require a different treatment strategy, as intramuscular benzathine PCN G, the typical formulation utilized for the treatment of primary, secondary, or latent stages of syphilis, does not achieve sufficient concentration levels in the CSF. Treatment of neuro, ocular, and otic syphilis therefore must be with IV aqueous crystalline PCN G which provides an appropriate treponemicidal level within these physiologic compartments [8]. Specific treatment with these diagnoses should be with IV aqueous crystalline PCN G in an 18 to 24 million unit dose given continuously or by six daily doses for 10 to 14 days per the Centers for Disease Control and Prevention [11]. A multidisciplinary team, as was utilized for this case, is recommended for patient evaluation and treatment and may include specialists such as maternal-fetal medicine, infectious disease, ophthalmology, neurology, otolaryngology, and neonatology. A diverse care team will promote proper workup and treatment and ensure that follow-up testing is performed in pregnant people with evidence of a disseminated syphilis infection.

The case report has limitations which include uncertainty regarding the pathophysiologic process responsible for the patient's symptoms: the osseous lesions versus dissemination of the syphilis infection to the maternal CNS. The report is also limited by the lack of supporting information available on rare complications and treatment of syphilis in pregnancy and whether this affects neonatal outcomes. The report has several significant strengths, highlighting that nonspecific symptoms in pregnancy may require a more judicious workup considering the increasing rates of syphilis worldwide. Lastly, the case highlights that the appropriate treatment for neurosyphilis is with aqueous penicillin as opposed to intramuscular penicillin G, the latter of which is utilized for straightforward secondary syphilis. The aqueous penicillin preparation is key to avoiding undertreatment of neurosyphilis in pregnancy, a serious concern given the significant risks to the fetus in an untreated infection.

Syphilis infections represent a significant, worldwide public health crisis, and as such, infection rates in pregnancy are concomitantly on the rise. While routine serologic testing for the infection is recommended at the initial prenatal visit, pregnant people remain at risk for infection acquisition later in gestation. Although providers should rescreen individuals who may be at higher risk of syphilis, the index of suspicion for a syphilis infection when clinical symptoms are atypical or unresolved with usual measures should remain heightened given the high stakes of congenital infection when treatment is not undertaken. Headache or other neurological symptoms can signify the onset of a disseminated Treponema pallidum infection and in rare cases can result in osteitis and neuro, otic, or ocular syphilis. Neuro, otic, and ocular syphilitic infection requires aqueous crystalline penicillin (as opposed to intramuscular penicillin G), but there is no agreement on the specific treatment for osteitis. Pregnancy and neonatal outcomes specifically associated with osseous, neuro, otic, and ocular syphilis also remain elusive at this time which requires the ongoing collection of cases and subsequent reporting.

## Data Availability

Data in support of this article is provided in the citations.
